# Spontaneous Diaphragmatic Hernia

**DOI:** 10.5811/cpcem.2018.5.38587

**Published:** 2018-07-16

**Authors:** Mark D. Darocki, Anthony J. Medak

**Affiliations:** University of California San Diego, Department of Emergency Medicine, San Diego, California

## Abstract

A spontaneous diaphragmatic hernia (SDH) occurs when intra-abdominal contents extend into the thoracic cavity through a defect in the diaphragm after a sudden increase in intra-abdominal pressure. SDH is one of the rarest surgical emergencies with less than 30 reported cases in the literature.^1^,^2^ In our case a 94-year-old female presented to the emergency department in respiratory distress with unilateral breath sounds and was diagnosed with a SDH. The only treatment option for a SDH is surgical.^3^,^11^ However, nasogastric tube decompression of the gastrointestinal tract and supplemental oxygen can be used to alleviate symptoms until definitive operative management is performed.

## INTRODUCTION

A patient presenting to the emergency department (ED) in respiratory distress with unilateral breath sounds does not have an extensive differential diagnosis. In a trauma scenario a pneumothorax or hemothorax quickly comes to mind. It is taught that a chest tube should be inserted without further diagnostic testing. In the non-traumatic patient a spontaneous pneumothorax, pleural effusion, pneumonia, or airway foreign body are among the most likely etiologies. Spontaneous diaphragmatic hernia (SDH) is not often considered, and thus may be a missed diagnosis during initial evaluation.

SDH is one of the rarest surgical emergencies comprising less than 1% of all diaphragmatic hernias, with fewer than 30 reported cases in the literature.^1^,^2^,^4^,^5^ A SDH occurs when intra-abdominal contents extend into the thoracic cavity through a defect in the diaphragm after a sudden increase in intra-abdominal pressure. SDH has been attributed to physical exercise, labor and delivery, coughing, vomiting, or defecation^1^–^3^ Diaphragmatic hernia is a rare surgical emergency that usually occurs in the setting of trauma. Blunt trauma is more common than penetrating trauma, and the most common cause is a motor vehicle accident.^1^ The most common structures to enter the thoracic cavity are the stomach, colon, greater omentum, small intestine, spleen, and liver.^1^,^2^

## CASE REPORT

A 94-year-old woman with chronic obstructive pulmonary disease, hypertension, and breast cancer presented to the ED in respiratory distress. She reported dyspnea starting the night prior to presentation with no history of trauma. She was normothermic, had a normal heart rate and blood pressure, but was tachypneic and hypoxic to 88% on room air. Physical exam revealed significant accessory muscle use, no stridor, no jugular venous distention, normal heart sounds and diminished breath sounds in the left hemithorax.

**Image 1:**
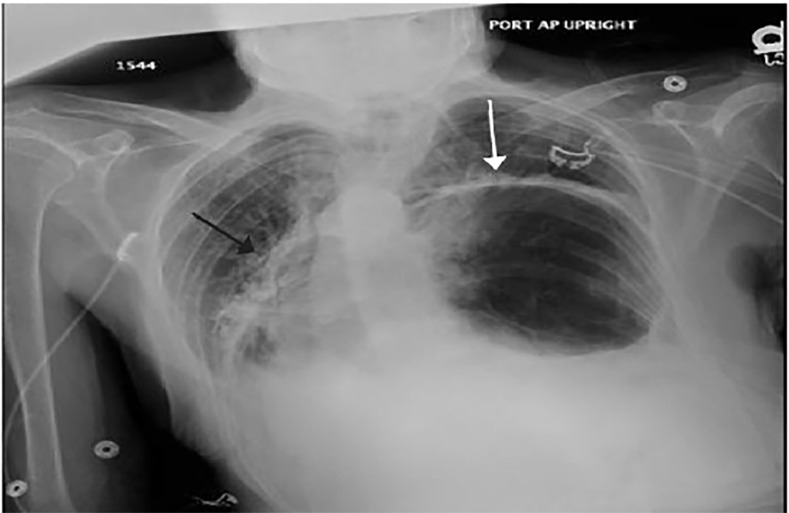
Anterior-posterior chest radiograph demonstrating intrathoracic gastrointestinal contents (white arrow). Note the shift of the mediastinum into the right hemithorax (black arrow).

While the nurse gathered a 14-gauge needle and a chest tube tray for needle decompression followed by tube thoracostomy, a bedside ultrasound was performed. The ultrasound showed bilateral pleural sliding without significant B-lines or effusion. Portable chest radiograph revealed that a large amount of intra-abdominal contents had entered the thoracic cavity resulting in a shift of the mediastinum (Image 1). A nasogastric tube was not inserted to decompress the bowel, as the patient declined to have this performed.

**Image 2:**
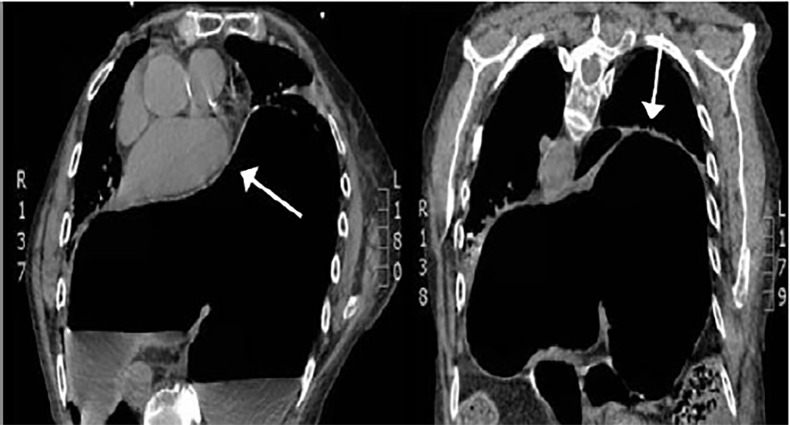
Computed tomography (CT) of the chest. Left: Axial view of a CT chest demonstrating gastrointestinal (GI) contents in the thoracic cavity with cardiovascular compression (white arrow). Right: Coronal view of a CT chest demonstrating GI contents in the thoracic cavity (white arrow).

We consulted the general surgery service, which recommended obtaining a computed tomography **(**CT) scan to further characterize the defect in the diaphragm (Image 2). The patient and her family members declined surgical intervention. She was admitted to the hospital to arrange home hospice care and was discharged within 24 hours. She died at home with her family three days after presenting to the ED.

## DISCUSSION

A SDH can be considered based on the history and physical exam, but imaging is required to make the diagnosis. It is important to consider SDH as part of an early differential diagnosis, as a delay in diagnosis increases the risk of strangulation, perforation, and pulmonary or vascular compression.^1^,^6^ Historical red flags include a known diaphragmatic defect,^6^ dyspnea preceded by a sudden increase in intra-abdominal pressure, and/or dyspnea in conjunction with vomiting. Patients commonly present with abdominal pain, chest pain, nausea, vomiting, and difficulty breathing.^1^,^5^ Physical exam findings include the absence of breath sounds, decreased breath sounds, or the presence of bowel sounds in the thoracic cavity.^6^

**CPC-EM Capsule**

What do we already know about this clinical entity?

A spontaneous diaphragmatic hernia (SDH) occurs when intra-abdominal contents extend into the thoracic cavity through a defect in the diaphragm after a sudden increase in intra-abdominal pressure.

What makes this presentation of disease reportable?

SDH is one of the rarest surgical emergencies with fewer than 30 reported cases in the literature.

What is the major learning point?

The diagnosis of SDH should be considered in patients presenting in respiratory distress with unilateral or asymmetrical breath sounds.

How might this improve emergency medicine practice?

Increased awareness of this rare diagnosis could prevent the unnecessary and potentially harmful placement of a chest tube in this patient population.

Diagnostic imaging options in the ED include chest radiograph, CT, ultrasound, magnetic resonance imaging, and upper gastrointestinal (GI) contrast studies. The most readily available options are radiograph, point-of-care ultrasound, and CT.^9^ Chest radiographs are diagnostic in only 25–50% of cases,^7^,^9^ and an estimated 66% of diaphragmatic hernias are missed on initial presentation.^8^ Common radiographic findings include the following: elevated left hemidiaphragm; blunting of the costophrenic angle; distortion of the diaphragm boarders; curling of gastric tube into the thorax; mediastinal shift; pleural effusion; or presence of air-filled GI structures in the thorax.^5^,^7^,^8^ Ultrasound can be complicated by scattering of the beam by the aerated lung and gas-filled intestinal structures, as well as acoustic shadowing from the ribs.^9^,^10^ CT is the most accurate imaging modality available in the ED. Depending on the location of the lesion, the accuracy of CT ranges from 50–78%.^9^

The most accurate method of making the diagnosis is via exploration in the operating room.^13^ However, even this is not 100% accurate.^14^ The only treatment option for a diaphragmatic hernia is surgical.^3^,^11^ However, supplemental oxygen as well as a nasogastric tube to decompress the GI tract can be used to alleviate symptoms until definitive operative management is performed.^4^ In our case, the bedside ultrasound exam did not confirm the diagnosis of SDH, but it did make the alternative diagnoses of pneumothorax or large pleural effusion much less likely. This prevented the patient from undergoing unnecessary needle decompression followed by tube thoracostomy, and the pain and morbidity associated with these procedures.

## CONCLUSION

Spontaneous diaphragmatic hernia is a rare diagnosis that is often missed on the initial patient encounter. While the treatment is surgical, therapeutic measures can be taken in the ED to alleviate symptoms and suffering. The utility of bedside ultrasound in the diagnosis of SDH is yet unproven. However, in our case it rapidly excluded a pneumothorax and prevented the unnecessary placement of a chest tube in an elderly woman.

Documented patient informed consent and/or Institutional Review Board approval has been obtained and filed for publication of this case report.
